# Developing an inclusive approach to fNIRS research in women of color

**DOI:** 10.1117/1.NPh.13.S1.S13003

**Published:** 2025-09-11

**Authors:** Stacey L. Gorniak, Luca Pollonini

**Affiliations:** a University of Houston, Department of Health and Human Performance, Houston, Texas, United States; b University of Houston, Department of Engineering Technology, Houston, Texas, United States; c University of Houston, Department of Electrical and Computer Engineering, Houston, Texas, United States; d University of Houston, Department of Biomedical Engineering, Houston, Texas, United States

**Keywords:** wearable neuroimaging, exclusionary practices in neuroscience, phenotype bias, phenotypic exclusion, inclusion

## Abstract

**Significance:**

Acknowledgement of a lack of inclusivity in human subjects–focused wearable neuroimaging research has emerged in recent years due to a number of common practices. Women of color are most negatively impacted by this systemic exclusion in wearable neuroimaging research, specifically with respect to functional near-infrared spectroscopy (fNIRS) research.

**Aim:**

We aim to demonstrate the effectiveness of holistic and inclusive practices in the recruitment and retention of women of color in an fNIRS research study.

**Approach:**

The development of inclusive approaches involves consideration of study design elements, study participant recruitment and retention, pre-fNIRS session assessment considerations, and adjustments to data collection practices during fNIRS assessment. Approaches taken in each of these study stages are described.

**Results:**

All participants remained enrolled and participated in the study through the online survey (S0), first in-person session (S1), and second in-person (fNIRS) session (S2), leading to a study retention rate of 100%.

**Conclusions:**

The inclusion of four key recommendations in addition to prior recommendations can help reduce exclusionary practices in human subjects–focused fNIRS research, particularly in recruiting and retaining women of color. Improvement of this approach is expected through iterative modifications and the inclusion of additional holistic practices.

## 1 Introduction

Wearable transdermal neuroimaging techniques such as functional near-infrared spectroscopy (fNIRS) and electroencephalography (EEG) are cost-effective and noninvasive neuroimaging tools that are rapidly gaining diffusion in academia and research & development industries alike. fNIRS is a portable and affordable alternative to functional magnetic resonance imaging (fMRI) for investigating cortical hemodynamic activity. fNIRS is often used to overcome limitations encountered in the use of fMRI such as potential injury during scanning due to implanted metals, experimental and physiological adverse effects of noise, movement artifacts, and lack of ecological validity of experiments.^1^^,^^2^ The reliance on detecting blood oxygenation level dependent (known as BOLD) in fMRI provides additional limitations as fMRI is only sensitive to deoxygenated hemoglobin (HbR, due to its strong paramagnetization^3^), whereas the oxygenated hemoglobin (HbO) aspect of the hemodynamic response is diamagnetic and thus undetected by fMRI.^4^ By contrast, both HbR and HbO are detectable using fNIRS—an important advancement that has assisted researchers in better understanding functional cortical activations.^5^^–^^8^ EEG is the primary technique for measuring brain electrophysiology, i.e., the electrical activity of neurons as a function of behavior, cognition, and other voluntary and involuntary tasks. Similar to fNIRS, EEG is wearable and lightweight and therefore apt for use in naturalistic studies.

Despite the advantages of fNIRS over fMRI, scientists encounter several challenges that have led to exclusionary practices in human subjects–focused fNIRS research. Lack of optode/sensor (i.e., emitter and/or detector) adherence to the scalp,^9^^,^^10^ presence of hair between the sensors and the scalp,^10^^,^^11^ volume and density of hair present, increased adiposity layer between the sensors and cortical tissue,^12^^,^^13^ and increased melanin content of the skin^9^^,^^11^ are some of the most common challenges encountered by fNIRS researchers who undertake human subjects–focused research. Some of these issues can be resolved using standardized practice in the placement of fNIRS optodes, whereas other challenges remain as inherent features of human variability across study participants and patient samples. In recent years, a handful of labs have attempted to overcome these challenges, including emerging work in global populations.^14^^–^^19^

**Fig. 1:**
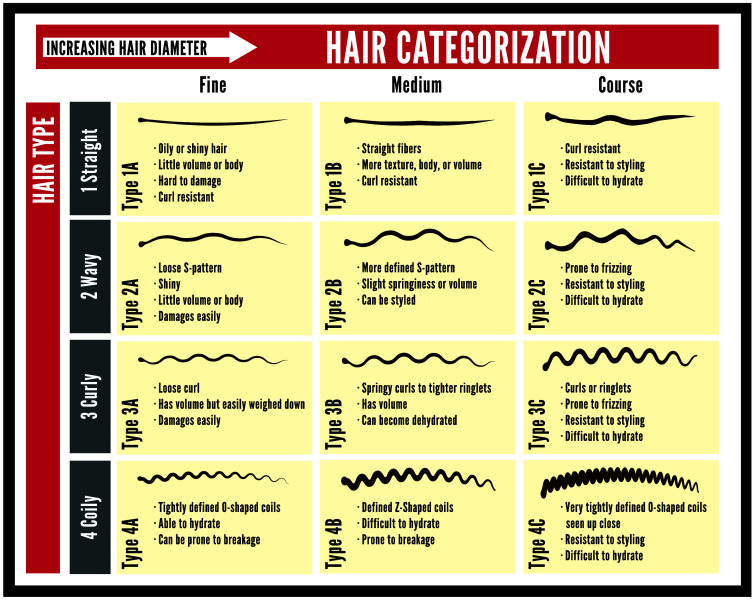
Illustration of the Andre Walker hair typing system.

One particularly notable challenge to the wearable neuroimaging community is when a participant/patient presents to the fNIRS session with dense hair or large amounts of hair. Previous studies involving the use of wearable neuroimaging devices, including fNIRS and EEG, have historically primarily included individuals with low to no amounts of hair, potentially leading to the inclusion of predominantly males in adult-focused studies. For fNIRS and EEG studies, including individuals with hair, a persistent practice regarding hair phenotype has emerged.^11^^,^^20^^–^^23^ In this practice, hair phenotypes that result in low hair volume, commonly known as Andre Walker hair types 1A and 1B (Fig. 1), have been included in both fNIRS and EEG studies.^11^^,^^22^ Andre Walker hair types 1A and 1B are also associated with low levels of skin pigmentation. Hair phenotypes types outside of Andre Walker hair types 1A and 1B (and consequently individuals with higher levels of skin pigmentation) have been excluded, as such hair types are “too voluminous” to be considered compatible with fNIRS and EEG studies. For individuals with voluminous hair types or hairstyles, attempts to improve fNIRS and/or EEG measurement outcomes are often abandoned—even in clinical environments. In recent years, a small number of labs have attempted to overcome hair type challenges using braiding techniques to improve compatibility with fNIRS and/or EEG measurement.^24^^–^^26^ Despite these efforts, these approaches have not gained traction.

As a direct result, the data collected to better understand: (1) how the brain works and (2) how to detect, diagnose, and treat a number of neurological and mental health conditions based primarily on data collected from men with little to no hair or in individuals with specific hair phenotypes associated with low levels of skin pigmentation—reinforcing well-established non-Hispanic White male-centric research phenotypical biases that inform clinical practices and development of clinical treatments, including clinical trials.^27^^–^^29^ The result is a form of systemic exclusion that reduces the prospect for specific populations (i.e., persons of color) benefiting from human-based neuroscience research.^30^ In particular, women of color are most negatively impacted by this systemic exclusion in wearable neuroimaging research.

Consequently, women and persons of color will be disproportionately impacted when fNIRS technologies transition to regular clinical use for diagnostic and monitoring purposes, consistent with historic practices in the low representation of women of color in clinical research.^31^ To counteract this, our group has been working to develop holistic strategies to be more inclusive in human subjects–based fNIRS research—specifically for women of color. In this paper, we present key aspects of our holistic inclusive approach coupled with demographic and retention data as a result of using the outlined approach in the target population—women of color 30 to 65 years of age residing in a racially diverse metropolitan area in the United States.

The holistic approach developed by our collaborative labs for human-based fNIRS studies was informed from a variety of sources, including discussion of exclusionary practices in neuroscience,^11^^,^^20^^,^^22^^,^^30^^,^^32^ the development of inclusive approaches to EEG data collection,^21^^,^^24^ and approaches used in qualitative sciences.^33^^,^^34^ Development of these inclusive approaches involves consideration of study design elements, study participant recruitment and retention, pre-fNIRS session assessment considerations, and adjustments to data collection practices during fNIRS assessment. We acknowledge that this paper is an initial and incremental step in improving inclusionary practices in human subject–based fNIRS research.

## 2 Materials and Methods

### 2.1 Acknowledgement and Understanding of the Historical Problem

Digesting the evidence is the key first step to understanding how current practices in neuroimaging studies and treatment informed by neuroscience reinforce exclusionary-based practices. This consists not simply of reading papers but also of reflecting on the practices of one’s own lab and how they may contribute to and perpetuate a history of exclusionary practices in neuroscience. This reflection includes active consideration of how such practices developed over time and through interaction with both the scientific community and participants. It also includes considering how these practices impact the life cycle of a study—starting with study creation and design, participant recruitment strategies, participant testing protocols, and ending with dissemination of study findings.

Concurrent with digestion and reflection, interacting with groups involved in community-based participatory research (CBPR) approaches such as Black in Neuro^32^ is vital. Through these interactions with experts in population-specific experiences, researchers can not only better understand the persistence of exclusionary practices in neuroscience but also learn about and actively engage with researchers globally who are developing inclusive methods and practices in neuroscience and neuroimaging. Through these activities, researchers can begin to consider and address how practices they engage in may contribute to exclusionary practices in neuroscience.

### 2.2 Addressing Barriers to Participation and Study Design Considerations

One approach to address exclusionary practices in fNIRS research is to utilize CBPR approaches when considering the creation and design of an experimental study. CBPR is defined as an approach that includes equitable partnership with the communities and populations affected by and knowledgeable of the circumstances of interest.^35^ Leverage of CBPR to inform research design, implementation, and results dissemination is mutually beneficial to scientists and the larger community.^35^ Investment of both scientists and community partners in the CBPR process can ultimately be utilized to collaboratively disseminate and share resources, ideas, and expertise.^35^

Our group utilized CBPR approaches for the project described in this paper in two ways. Our initial intentional CBPR interactions involved participation in the 2022 Black in Neuro Week Conference, which included both a panel discussion and a journal club seminar focused on exclusionary practices in wearable neuroimaging modalities. Participation in these events informed our research team on the historical context of exclusionary practices and the development of counter practices.

Our second intentional CBPR interaction was with the University of Houston’s (UH) Helping Everyone Achieve a Lifetime of Health (HEALTH) Center for Addictions Research and Cancer Prevention Research Centers in Minority Institutions (RCMI) Specialized Center. As required by the National Institutes of Health’s mandatory RCMI guidelines, the UH HEALTH RCMI is centered on the investigation of health conditions that disproportionally impact underrepresented minorities and other underserved and/or marginalized communities.^36^ As a means to facilitate investigation, the UH HEALTH RCMI provides numerous shared resources that are centralized (commonly known as “research cores”) to stimulate and increase research productivity. The Community Engagement Core within the UH HEALTH RCMI hosts a Community Research Advisory Board (CRAB)^37^^,^^38^ that works with investigators to provide community-based knowledge, experience, and insights into investigator-initiated research. We met with the UH HEALTH RCMI CRAB twice over the course of 6 months to interact with community members to help identify barriers to study participant recruitment, participation, and retention for our fNIRS studies. The UH HEALTH RCMI CRAB offered invaluable insights and advice to our research team regarding the recruitment and retention in the target population of our study (i.e., women of color).

In our discussions with the UH HEALTH RCMI CRAB, the following specific barriers were identified and discussed:

1. Availability for testing during typical business hours (Monday to Friday, 9 am to 5 pm): Due to work constraints, many individuals in our target study population in the United States (US) would not be able to attend in-person fNIRS sessions scheduled during typical US business hours.^39^ This is significant as most research and clinical labs are only operational during typical US business hours.
2. Conflict with childcare and/or eldercare responsibilities: As women bear a disproportionate amount of responsibility in both childcare and eldercare responsibilities in the US, a significant proportion of our target study population may not be able to participate in in-person fNIRS sessions during typical US business hours and beyond.^40^^,^^41^
3. Use of public transportation: Consistent with other large metropolitan areas in the US, persons of color in the greater Houston metropolitan area tend to use public transportation as their primary means of transportation.^42^^,^^43^ The location and distance of public transportation stops from fNIRS labs may additionally contribute to low study participation rates in our target population.
4. Development of recruitment materials and approaches appropriate for the target population: A significant history of marginalization and mistreatment of women of color in medical research science^44^^–^^48^ has led to mistrust of the scientific community by communities of color.^49^^,^^50^ To build trust with these communities, cultural considerations in study design, participant recruitment, participant retention, and participant compensation should be discussed and designed interactively with the communities that consist the target population.

To address issues #1 and #2 raised by the UH HEALTH RCMI CRAB, our study staff worked together collaboratively to develop a flexible schedule each semester to accommodate early morning (7 am or earlier), after work (after 5 pm, Monday to Friday), and weekend testing sessions (Saturdays and Sundays) as much as possible.

To address issue #3, our study staff offered free bus/rail system daily passes to all participants (Houston METRO Day Pass), which allowed free unlimited transfers within the metropolitan transportation network. Houston METRO has both bus and rail stops within 0.2 miles (320 m) from our labs. For participants who have limited mobility and are unlikely able to travel independently between the nearby bus/rail stops and the labs, Houston METRO Lift passes were provided. Houston METRO Lift service is an Americans with Disabilities Act accessible, shared-ride paratransit service with customized curbside service scheduled as per the needs of riders. The Houston METRO Lift drop-off location for our labs is in front of the building that houses the labs. Participants who instead preferred to drive to the lab for their in-person sessions were provided free surface-lot parking located immediately outside of the building that houses the labs.

To address issue #4, we discussed our study design and objectives interactively with the UH HEALTH RCMI CRAB on two occasions, scheduled 6 months apart. During these discussions, the UH HEALTH RCMI CRAB recommended that our recruitment materials be redesigned specifically for our target population. We engaged with the UH HEALTH RCMI graphic designer on multiple instances for this task. This collaboration produced two redesigned recruitment flyers for our study. In addition, the UH HEALTH RCMI assisted our lab with participant recruitment by distributing our redesigned recruitment flyers to community-based organizations that primarily involve women of color. Our redesigned recruitment flyers were also distributed by UH HEALTH RCMI staff and key community partners at public health promotion events attended primarily by women of color.

### 2.3 Pre-fNIRS Assessment Protocol

Prior to any fNIRS experimental activity, our lab has undertaken several efforts to increase study retention between recruitment efforts and arrival at the lab for in-person fNIRS testing sessions. In each of the subsections below, we expand on the steps taken.

#### 2.3.1 Development of a specialized fNIRS cap for use by women and persons of color

**Fig. 2:**
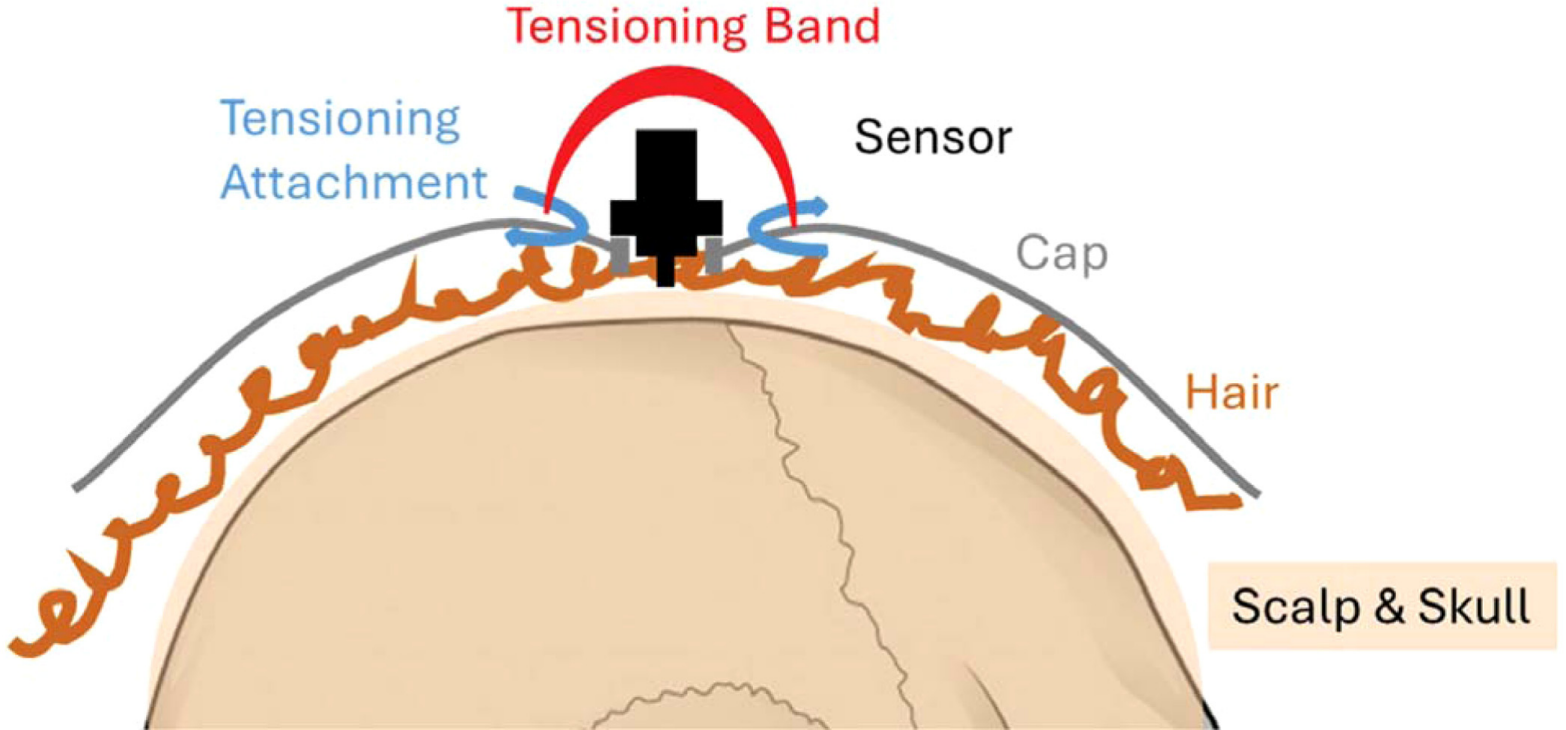
Conceptual illustration of the premise of TensiCap, consistent with Patent Cooperation Treaty Application (PCT US2025/024299).^51^

To improve fNIRS measurement outcomes during fNIRS measurement in women and persons of color, our lab has developed a device to improve adherence of wearable neuroimaging sensors (i.e., fNIRS optodes/sensors and EEG electrodes) to the scalp,^51^ known in our lab as TensiCap. The device can be used for any wearable neuroimaging system (i.e., nonspecific to any maker/manufacturer) and is compatible with standardized fNIRS and EEG sensor layouts as well as nonstandard (lab customized) sensor layouts. The configuration of the device elements can be customized for each participant to accommodate hairstyles such as braids, dreads, and locs. In this particular study, TensiCap v2.0 was utilized during data collection for this project. A conceptual illustration of the premise of TensiCap, consistent with Patent Cooperation Treaty Application (PCT US2025/024299),^51^ is shown in Fig. 2.

#### 2.3.2 Single point of lab contact, schedule of interactions, and integration of phenotype questionnaires

To streamline communication with study participants, the individual designated as the primary investigator (PI) on the recruitment materials and the informed consent document was the individual whom participants communicated with prior to their arrival in the lab. This individual was responsible for scheduling all in-person testing sessions and distributing directions to the lab. In addition, this individual was the first person each study participant met for their first in-person session. The contact information for this individual (including name, credentials, email address, and phone number) was provided via email shortly after a participant qualified for the study.

Ahead of in-person fNIRS sessions, study participants were required to complete all surveys and questionnaires via an online survey portal (Qualtrics, Seattle, Washington, United States) to maximize the amount of time spent in the lab on fNIRS-focused tasks. Included in this online survey (considered Session #0, S0) were self-report questions regarding Andre Walker hair type and Fitzpatrick skin scale, consistent with self-report phenotype tracking recommendations for inclusivity assessments.^11^^,^^22^ Survey questions regarding Andre Walker hair type and Fitzpatrick skin scale utilized visual representations of each hair and skin phenotype to aid participant self-report. Visual representations were selected from reliable online resources. An example of Andre Walker hair type scale can be found in Fig. 1. Study participants of all hair types were eligible for participation in this study; no participants were excluded based on hair type. Information regarding inclusion/exclusion criteria can be found in Sec. 2.6.

Once all online study surveys were completed (S0), participants were asked to arrive at the lab for one in-person testing session (session #1 (S1), ∼1  h long) that did not include fNIRS testing. At S1, participants’ first in-person contact with the lab was with the named study PI. The pre-fNIRS session included anthropometry measurement, body composition measurement via dual energy X-ray absorptiometry (also known as DXA), and a venous blood draw. These measurements were for covariate assessments for our study. At the end of this pre-fNIRS session (S1), participants underwent the items listed below in Secs. 2.3.3 and 2.3.4.

#### 2.3.3 Introduction and discussion of the fNIRS cap and respect for participant hair considerations

After completion of the pre-fNIRS session measurements in S1, study personnel used an approach known as empathetic neutrality to introduce the fNIRS technology to be used in the next in-person session. Participants were shown an image of the fNIRS cap (TensiCap v2.0) to be used in the next (fNIRS-focused) in-person testing session (session #2 (S2)). The image was a photograph of the study PI (SLG) wearing the actual fNIRS cap from the lab. Study personnel then explained that the optical elements located on the cap (i.e., the optodes) would need to be touching the scalp throughout the fNIRS testing session. Study personnel and participants then engaged in active discussion regarding how the cap would be placed on the participant’s head and how hair may need to be moved to accommodate the optodes in an optimal position for data collection.

Study personnel were instructed to utilize active listening techniques regarding participant questions and concerns regarding the fNIRS cap and fNIRS sessions and to adopt a nonjudgmental attitude in interpreting participant questions and concerns regarding fNIRS. Validation and reflection of participant concerns regarding fNIRS sessions, particularly regarding hair considerations, were encouraged by study personnel. During these discussions, study personnel were not to make any assumptions regarding participant hair and potential challenges in collecting fNIRS data. In addition, study personnel were not to touch the participant hair unless invited by the participant. During discussions, study personnel would gently indicate to all participants that materials such as wigs, extensions, hairpins, and weaves (also known as sewn-in extensions) would not be able to be worn by participants during fNIRS sessions, independent of hair type, style, or length. As a response to the introduction of how hairpieces (e.g., wigs, extensions) interfere with fNIRS sessions, participants typically responded with an assessment of their current hairstyle as well as current and/or planned use of wigs and/or extensions in the upcoming weeks.

#### 2.3.4 Flexibility in fNIRS scheduling

During these hair-centric discussions, study personnel were encouraged to ask questions regarding how hairstyle and upcoming hair appointments impact participant availability for in-person fNIRS sessions. In some cases, participants presented to the lab with braids, weaves, dreads, or locs during S1. During hair conversations at the end of S1, some participants would indicate that they would prefer to attend their fNIRS session when their braids or weaves were removed and before the next set of braids or weaves was installed. Other participants would discuss their braiding or loc twisting schedule with study personnel. In these cases, participants would be asked to schedule their in-person fNIRS session (S2) for a day or two before “wash” day. This was requested by study personnel as the days before wash day are when the braids, dreads, and locs are at their loosest and able to be moved/manipulated to optimize optodes’ adhesion to the scalp. For participants with voluminous hairstyles that did not use braids, dreads, or locs, most participants opted to arrive at the fNIRS testing session with hairstyles that included blowouts or flat-ironed hair.

Accommodation of scheduling the in-person fNIRS session (S2) for each participant considered any indicated hair-centric scheduling considerations as well as scheduling conflicts as indicated in Sec. 2.2.

### 2.4 During fNIRS Assessment

During fNIRS assessments, our lab has undertaken several efforts to improve study participant comfort and ensure completion of all fNIRS protocols during in-person fNIRS sessions. The fNIRS session lasted ∼2.5  h and consisted of tests of cognitive and motor functions while the participant was seated. Information regarding fNIRS montage,^7^^,^^8^ cognitive testing,^7^ and motor function testing^8^ can be found documented in our prior work. In addition, cognitive testing via a language production task (picture naming) and a nonverbal switching task (shape-color task), as described in Ref. 52, was also included in the cognitive testing battery.

#### 2.4.1 fNIRS cap fitting, improving sensor adhesion, and consideration of participant comfort

To improve coupling between the participant scalp and optodes in this study, study staff took a multistep approach. First, study staff assisted participants in donning the TensiCap v2.0. Then, study staff used internally illuminated plastic ear curettes with soft silicone tips to gently move any hair, braids, dreads, or locs within the optode path. Afterward, optodes were placed into the TensiCap v2.0 and study staff adjusted the TensiCap to accommodate any gaps between each optode and the scalp. Study staff inquired multiple times regarding the participant comfort. Participants were asked to indicate if any of the actions taken by study staff during TensiCap placement were uncomfortable. In the event that discomfort was expressed by a participant, study staff would stop the given activity and modify their approach according to participant comfort.

### 2.5 Institutional Approvals

In accordance with the Declaration of Helsinki, participants provided informed consent according to the regulations established by the Institutional Review Board at the University of Houston (STUDY00004312).

### 2.6 Participant Inclusion and Exclusion Criteria

Right-handed study participants aged 30 to 65 years of age who were assigned as female at birth and self-identified as a minority/underrepresented person (i.e., African American, Hispanic, Latina, Native American, etc.) were recruited for this study. Women who identified as any group other than White Non-Hispanic were permitted to enroll in the study, as per IRB and sponsor-approved criteria. Study participants were excluded if they reported a history of: type I diabetes, recent major injury or surgery, prior major surgical intervention to the upper extremity, limb amputation, chemotherapy-induced peripheral neuropathy, or other neurological and/or musculoskeletal disorders (cerebrovascular accident (including mini-stroke), Parkinson disease, Huntington’s disease, polio, multiple sclerosis, stroke, traumatic brain injury, carpal tunnel syndrome, rheumatoid arthritis, Lyme disease, Scleroderma, Monoclonal Gammopathy of Undetermined Significance (MGUS), Paraproteinaemic Demyelinating Neuropathy (PDN), Myasthenia Gravis), or hereditary or compression neuropathies.

Participants who presented with a history of prediabetes or type II diabetes were permitted to enroll in the study as the aim of the larger study was to understand how metabolic state influences overall health, sleep, and memory in women of color. We have included the recruitment flyers used for this study as Supplementary Material. Scheduling data for the overnight sleep session associated with this study was not included in the current paper as scheduling and sleep study testing were coordinated by a subcontracted clinical site; however, retention of participants in the sleep study portion of the project remained at 100%.

Recruitment of study participants was from the greater Houston metropolitan area. The greater metropolitan area is composed of over 4.5 million residents from a broad spectrum of ethnic backgrounds. The racial distribution of the greater Houston community (Harris County, Texas, United States) according to the 2020 census is: 46.8% White, 22.7% African American/Black, 6.8% Asian, 0.5% American Indian/Alaska Native, 0.1% Native Hawaiian/Other Pacific Islander, and 10.8% Multiracial.^53^ The ethnic distribution is 44.5% Hispanic or Latino. The current population of Houston, Texas, roughly reflects the anticipated national census results in 2060.^53^^,^^54^

Participants were compensated for their participation in each aspect of the study at a rate of ∼$20 an hour in the form of Amazon gift cards. Compensation for each session is indicated in Sec. 3.2.

## 3 Results

### 3.1 Participant Demographics

Fifty (50) women between the ages of 30 and 65 years old (mean±SD=46±9 years) who self-identified as belonging to an underrepresented group were recruited and enrolled to participate in a study on health and memory in women of color. Self-reported race and ethnicity were collected consistent with National Institutes of Health standard reporting forms.^55^ Sample demographics, including age, body mass index, number of years of education, and number of languages spoken, participant-reported race, ethnicity, Andre Walker hair type, and Fitzpatrick skin type, can be found in Tables 1–4. Handedness was confirmed by the Edinburgh Inventory,^56^ ranging from a laterality quotient (LQ) of −100 (strong left-handedness) to +100 (strong right-handedness). Participants had an LQ average of +94.

**Table 1:** Participant age, body mass index, and educational background demographics.

	Mean or count	SD	Range
Age (years)	46	9	[30, 65]
Body mass index ($\mathrm{kg}/m^{2}$)	30.4	6.6	[20.7, 56.2]
# Years of education (years)	17	3	[12, 22]
# Years mother’s education (years)	13	4	[2,18]
# Languages spoken by participant	2	1	[1, 4]
# Monolingual participants	28	—	—

**Table 2:** Participant-reported race and ethnicity.

	Not Hispanic or Latino	Hispanic or Latino	Total
American Indian or Alaska Native	0	0	0
Asian	2	1	3
Native Hawaiian or Other Pacific Islander	1	0	1
Black or African American	25	2	27
White	0	14	14
More than 1 race	1	4	5
TOTAL	29	21	50

**Table 3:** Participant-reported Andre Walker hair type.

Hair structure	Hair diameter(smallest to largest)				Total
	Fine	Medium	Coarse	Not reported	
Straight hair (type 1)	5	1	0	1	7
Wavy hair (type 2)	2	7	1	3	13
Curly hair (type 3)	3	3	4	3	13
Coily hair (type 4)	0	5	10	1	16
Not reported	0	0	1	0	1
Total	10	16	16	8	50

**Table 4:** Participant-reported Fitzpatrick skin pigmentation scale level.

Skin type	Count
I (Ivory)	2
II (Fair or Pale)	2
III (Beige or fair with golden undertones)	10
IV (Olive or light brown)	14
V (Dark brown)	17
VI (Deeply pigmented dark brown to darkest brown)	5
Not reported	0
Total	50

### 3.2 Participant Scheduling Data

**Fig. 3:**
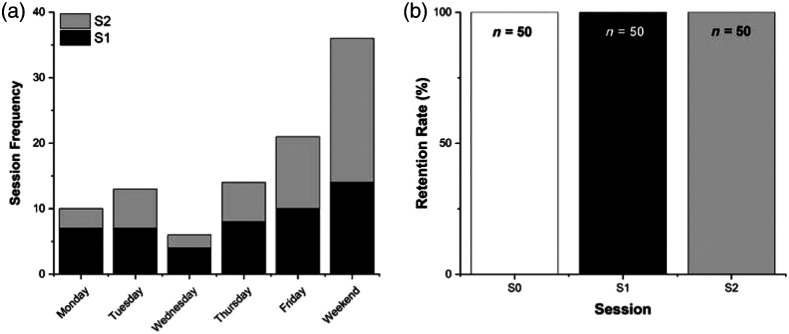
Session scheduling and retention data. (a) Distribution of session 1 (S1, in black) and session 2 (S2, in gray) across days of the week. S1 was a 1-h in-person visit without fNIRS assessment. S2 was a 2.5 h in-person visit with fNIRS assessment. Sessions on Saturdays and Sundays were combined in this figure as both days are considered outside of typical US business days. (b) Retention of study participants through each session. The total number of study participants (n) was 50 for each session.

All 50 study participants attended in-person sessions at the UH main campus. All study participants participated in one online-only session to complete study survey items (S0, compensation = $10 Amazon gift card) and one in-person pre-fNIRS session on campus (S1, approximately 1 h long, compensation = $20 Amazon gift card), held 1 week prior to their in-person fNIRS testing session. S0 occurred at the convenience of study participants. All S1 sessions were scheduled to begin between the hours of 7 am and 10 am as blood-based biomarkers of interest measured from the blood sample taken in S1 exhibit diurnal fluctuations. All in-person fNIRS testing sessions (S2) lasted ∼2.5  h (compensation = $60 Amazon gift card) and were scheduled at the time of day and on the day of the week most convenient to study participants. Figure 3(a) indicates the scheduling frequency of both S1 and S2 with respect to the day of the week; sessions on Saturdays and Sundays were lumped together as “weekend” as sessions on these days are considered outside of typical business days that most research and clinical labs utilize. Study participants most frequently requested S1 (n=14) and S2 (n=22) to be scheduled on weekend days (Saturdays and Sundays), followed by Fridays as the most frequent days for S2 scheduling (n=11).

With respect to time-of-day S2 scheduling, most sessions occurred between the hours of 8 am and 5 pm (n=46). Three (3) study participants requested an early start for S2 on a weekday as early as 7 am. One (1) participant requested a late start for S2 on a weekday, as late as 6 pm. The most frequently cited reason for testing sessions scheduled over the weekend or on a weekday outside of typical business hours was work responsibilities/schedule, followed by childcare responsibilities.

Of the 50 participants enrolled in this study, three participants requested METRO Day Passes to attend in-person testing sessions. The remaining 47 participants utilized the free surface-lot parking provided.

All participants remained enrolled and participated in the study through the online survey (S0), first in-person session (S1), and second in-person (fNIRS) session (S2), leading to a study retention rate of 100% from S0 all the way through the completion of S2, shown in Fig. 3(b).

## 4 Discussion

As women of color are the population at most risk for experiencing systemic exclusion in human-based neuroscience research and resultant clinical practices, our lab has worked to develop holistic strategies to combat this issue. We presented key aspects of our holistic and inclusive approach along with demographic and retention data in our target population—women of color 30 to 65 years of age. As evidenced by our data, we have had significant success in recruiting and retaining women of color in our human-focused fNIRS studies due to the holistic and inclusive approach described above. The development of inclusive approaches to fNIRS involves a combination of changes to researcher behaviors prior to study design, as well as changes in approaches to pre-fNIRS assessments and during fNIRS assessments. Although all aspects of our approach contributed to the 100% recruitment and retention rate of our study, we hereby discuss key aspects of our approach that had the largest impact as well as recommendations for labs that are considering revising their recruitment and retention approaches to counteract exclusionary practices in human subjects–based neuroscience research.

### 4.1 Engagement with Community Research Advisory Board (UH HEALTH RCMI CRAB)

Key aspects of our recruitment and retention efforts were direct outcomes of engaging in CBPR efforts with the UH HEALTH RCMI CRAB. Our work with a CBPR group located in our local community (and inclusive of our target population) assisted our group not only by informing research design and modifying our lab’s approach to implementation of this project but also by recruiting our target population using culturally appropriate materials and approaches. This collaborative approach to our research project not only reinforced investment of our research group in the community but also helped community receptiveness to the project and participation in the project itself. Upon conclusion of the study, most of our study participants expressed appreciation for the chance to participate in our research study as well as appreciation for the overall focus of the research project.

This appreciation was reflected in a way that we did not expect, such that more than one half of our study participants told other individuals in their families and social circles about the project. This word-of-mouth communication regarding our study led to an unanticipated form of snowball recruitment for our study. Most studies that utilize snowball recruitment methods do so intentionally to recruit members of underserved populations that are typically difficult to reach and engage in research. Upon reflection of the approach used in this study, our culturally sensitive approach, formed collaboratively with the UH HEALTH RCMI CRAB, was consistent with techniques used by some researchers employing snowball recruitment methods,^57^ with one major exception. In traditional snowball recruitment designs, researchers ask participants for contact information of one or more potential future participants. We did not take this approach in our study. Instead, the snowball recruitment variation that occurred in our study was that current participants provided contact information of our single point of contact person to interested individuals in their social circles and family networks who shared characteristics such as race, ethnicity, and age. These interested individuals then contacted the single point of contact person to see if they qualified for the study. One benefit of this unanticipated outcome was shortening the recruitment, enrollment, and data collection phases of the project to 9 months and 3 weeks, shorter than the 18 months originally anticipated for this phase of the study.

Although traditional snowball recruitment has been associated with reductions in sample variability,^57^ our data indicate a diverse sample of women who met study criteria participated in our study (Tables 1–4). Such inclusion of a diverse sample of women of color in human-focused fNIRS research shows how inclusionary practices integrated into the early phases of study design and creation can counteract a history of exclusionary practices in neuroscience research.

### 4.2 Flexible Scheduling for In-person Visits and Single Point of Contact

Another key aspect of our success in recruitment and retention of women of color in our study is the flexibility in scheduling in-person visits to the lab. Although study participants attended the 1-h pre-fNIRS in-person session (S1) scheduled from 7 am to 10 am fairly evenly across days of the week [Fig. 3(a)], the 1-h session was the maximum amount of time many women were able to attend on these days. Given that S1 had to occur early in the day to accommodate the required blood draw, the majority of participants attended this session prior to going to work for the day or early on a weekend day on which they did not have to work.

In terms of the 2.5-h fNIRS in-person session (S2), the majority of participants in our study indicated that they could not attend in-person sessions during the week due to employment and/or childcare responsibilities [Fig. 3(a)]. This accounted for the high percentage of S2 visits on weekend days. A high proportion of S2 sessions also occurred on Fridays in this study, as many companies within the greater Houston metropolitan area utilize what is referred to as a “9/80” work schedule. This schedule is a compressed work schedule in which employees work 4 days a week every other week, such that employees work a total of 80 h over 9 workdays, making the 10th workday (typically a Friday) an extra day off. Several of our participants noted that this “free” Friday was when they attended their S2 session.

In addition, S2 was scheduled at the convenience of many study participants with respect to hair care appointments, which frequently occurred on Fridays or Saturdays. Participants who wore braids, dreads, locs, and weaves appreciated the ability to schedule S2 with respect to their haircare needs.

By utilizing a single point of contact approach with this study, it allowed for direct and clear communication channels between the study personnel and participants, such that participants knew: (1) who would be reaching out with details regarding the study and (2) who they should contact if they had questions or needed to reschedule a session. This helped reduce confusion for participants, particularly those who had to navigate scheduling challenges to participate in the study. This approach was used to foster openness and transparency in the research process—both of which are particularly important to communities that have been marginalized in biomedical research.^58^ In support of this approach, the single point of contact model is frequently used and valued by patients and their families when managing care in clinical settings and clinical trials.^59^^–^^61^

Aside from the specific experimental requirements and needs of our study, fNIRS is a generally portable technique that opens opportunities to home-based experimentation that, in turn, may further facilitate scheduling, eliminate mobility/transportation burdens, and reduce participant–scientist barriers due to educational and cultural gaps as well as intimidating clinical/scientific settings. The latest generation of fNIRS instruments are fully wearable and can be operated from a laptop, and transportation of experimental equipment (computers, stimulation/response devices, additional sensors) to the participants’ home may be feasible in certain circumstances. By contrast, privacy and safety issues may actually prevent home-based research altogether; hence, such a trade-off between participation benefits and limits must be carefully considered.

### 4.3 Conversations about Hair and fNIRS

In addition to the points discussed above, participants were very interactive in the portion of S1 in which the fNIRS device was shown and hair considerations were discussed. By adopting the use of empathetic neutrality in these discussions of hair with study participants, study personnel could better adapt the discussion of hair considerations for study participants. Using approaches in which assumptions regarding hair type or the use of hairpieces are absent, authentic communication between study participants and study personnel regarding hair considerations for fNIRS assessment could occur. Using validation and reflection of participant concerns regarding hair considerations helped to facilitate discussion of what to expect during fNIRS assessments. In addition, these conversations allowed participants to plan ahead in terms of their haircare both prior to and after fNIRS testing. This pre-experimental discussion of hairstyle and use of hairpieces by study participants is a critical element of the proposed holistic approach. These discussions are designed to prevent uncomfortable and/or awkward situations in which the participant is put in the undesirable position of either removing hairpieces during an in-person session or disenrolling from the study. By having these discussions with study participants prior to arrival for in-person fNIRS sessions, this avoids: (1) discomfort of both participants and study personnel, (2) lost time and effort for both participants and personnel, and (3) development of participant disaffection toward future participation in research. Although this form of interaction with participants does take some time, this form of participant education is key to: (1) ensuring participant preparedness for fNIRS imaging sessions and (2) maximizing potential for successful fNIRS imaging.

In the case of this study, these discussions were held in person at the end of S1. We understand that in-person visits with study participants may not be feasible for all labs—especially clinical labs. As an alternative, short video calls (≤15  min) or telehealth meetings using audio and video capabilities could be adopted by study and/or clinical personnel to demonstrate donning an fNIRS cap and how hair considerations relate to fNIRS measurement. Given the variability in hair type and hairstyles, we recommend one-on-one discussions utilizing both audio and visual aids with study participants to discuss how hair styles and hairpieces impact fNIRS measurement. This one-on-one interaction can be customized for each participant’s specific questions regarding fNIRS. In addition, these one-on-one discussions provide the opportunity to discuss scheduling flexibility that may be needed to accommodate hair styling appointments. Without these efforts in participant preparedness in our prior studies, study participants have presented to the lab in wigs or with sewn-in extensions. In these cases, fNIRS sessions had to be rescheduled or prolonged to accommodate such hair considerations—ultimately leading to a longer recruitment, enrollment, and data collection period than originally anticipated.

In addition to these conversations, our group utilized a novel headgear developed in our lab specifically for use by women of color.^51^ Although developing an ideal headgear for optimized coupling of optodes for specific populations is a challenging task, several individual tools are available to fNIRS researchers for facilitating inclusion in studies with diverse participants. For instance, many fNIRS instruments feature caps and optode holders, which allow height adjustment (manual or spring-loaded) and lateral adjustments of optodes, to optimize their landing in correspondence with hair partings or spots with reduced hair density (e.g., between braids). The use of an elastic over-cap fit on top of the fNIRS headgear is also an effective way to stabilize the optodes against the participants’ scalp as well as to block the ingress of ambient light underneath the optodes. In terms of ensuring participant comfort during testing, our approach can be followed similarly in other fNIRS labs, especially those with headgears that allow for optode removal and subsequent placement, as well as height or tension adjustments. Despite these technical features, the issue of headgear poorly fitting certain research participants is still far from being addressed. Specifically, different populations have different head skull morphologies,^62^^,^^63^ yet neuroimaging caps (used for fNIRS, EEG, or other techniques) are available in a single shape for the worldwide population. This factor significantly affects research involving African and Asian populations, especially on the posterior part of the head where the headgear tends to fit loosely. We therefore recommend a collaborative effort between researchers and manufacturers aimed at developing population-specific headgears for achieving a higher-quality and wider-representative fNIRS research.

### 4.4 Acknowledgement of Study Specific Characteristics

The modifications discussed in the prior paragraphs are generalized to provide a broad framework for improved recruitment and retention of women of color in fNIRS research. We acknowledge that there are characteristics of this particular study that may have positively influenced our recruitment and retention rates beyond what has already been discussed. For example, the focus of this particular study on the influences of metabolic health on sleep and memory, specifically on women of color, is a novel topic that has not traditionally garnered much scientific attention. The novelty of the project was very well received due to its focus on important intersectional issues impacting the local community.

We acknowledge that men of color also experience marginalization in neuroscience research; however, our sponsor (National Institute on Aging, NIA) funded this project that included only women of color. Given that federal funding has restrictions, which require recipients to maintain the scope of work, we were not able to include male subjects in the current study, which may also have impacted recruitment and retention rates.

In addition, the fNIRS montage used in this study involved 16 optodes and 16 detectors in a setup that has been used previously by our lab.^7^^,^^8^ The tasks used in this particular study were straightforward and easy for participants to understand.^7^^,^^8^ Our lab has a significant history in using these tasks, including visual aids to convey participant instructions prior to testing. More complicated fNIRS montages or tasks may increase participant burden and fatigue, leading to participant dropout.

### 4.5 Limitations

Although this paper is the first attempt from our group to document successful approaches to recruitment and retention of a research population that has been historically marginalized, we acknowledge several limitations to the current paper. Although other studies have focused specifically on technical solutions for improving signal-to-noise ratio (SNR) in fNIRS,^9^^,^^10^^,^^64^ our study complementarily explored broader strategies aiming at greater inclusivity and participation. As described above, our cap fitting solution was meant to improve fNIRS signals, but, unlike these other studies, investigating solutions to specifically improve SNR was beyond the scope of the current study. In addition, as our study was not designed to achieve or demonstrate SNR improvement compared with a normative group, we believe that reporting our SNR data would be an incomplete and perhaps misleading effort. We believe that this study complements well other existing technical efforts in fNIRS research, and a combination of all proposed approaches would result in more inclusive and higher-quality fNIRS research across the research community.

We also understand that racial and ethnic subgroup considerations are also important in study design. Our original study design included a larger sample size to account for these subgroup differences. However, a sponsor-led change in the funding mechanism provided to our group limited the possible sample size to n=50 participants for the current study. We do plan on assessing for trends in potential racial and ethnic subgroup differences in fNIRS data collected in our subsequent publications, in which the actual fNIRS data collected during this project are presented.

### 4.6 Conclusion and Recommendations

Our holistic approach to inclusivity in human subjects–based fNIRS research has resulted in improved recruitment and retention of women of color in studies conducted in our lab. Key recommendations from our group for development and refinement of recruitment and retention strategies include the following:

1. inclusion of local CBPR groups in study development, design, and recruitment efforts;
2. use of a single point of contact for communication with the lab;
3. flexibility in scheduling fNIRS assessments longer than 1 h, including availability of weekend imaging sessions;
4. interactive discussion of fNIRS assessment and hair considerations with participants ahead of arrival to the lab for fNIRS sessions.

Inclusion of these four key recommendations in addition to prior recommendations regarding phenotype tracking^9^^,^^11^^,^^20^^,^^21^^,^^24^ can combat exclusionary practices in human subjects–focused fNIRS research. We acknowledge that the approach described in this paper is an incremental first step in improving fNIRS practices—particularly for women of color. We anticipate improvement of this approach through iterative modifications and inclusion of additional holistic practices in future work.

## Supplementary Material

10.1117/1.NPh.13.S1.S13003.s01

## Data Availability

Data analyzed in this project will be available via Zenodo (https://zenodo.org/) upon publication. As public sharing of protected health information such as date of birth, date of diagnosis, dates of treatment, and other private medical history information pertinent to each participant may violate HIPAA and the Texas Medical Privacy Act, these data will not be shared to protect patient identity.
