# Supplementary material for: Developing an inclusive approach to fNIRS research in women of color
Source: Neurophotonics. 2025 Sep 11;13(Suppl 1):S13003. doi: 10.1117/1.NPh.13.S1.S13003 (PMC12425162; doi:10.1117/1.NPh.13.S1.S13003)
Supplement: Supplementary file 1 [file NPh_013_S13003_SD001.pdf]

WOMEN SLEEP & MEMORY STUDY

Are you eligible to participate?

We are looking for minority women aged 30-65 that are right-handed, will take an online survey, and make two visits to our lab.

The study consists of:

- Responding to verbal cues during manual tasks
- Testing your cognitive functioning and memory
- Undergo a blood draw and body scan
- Evaluate your sleep characteristics

Qualified Participants will receive an Amazon gift card for completing:

- Online survey - $10
- First lab visit - $20
- Second lab visit - $60
- Sleep study - $200

Totaling $290 for attending all study visits.

For information on eligibility for this study:

Contact us by phone at 713.743.4802
Email us at sgorniak@uh.edu

SCAN HERE!

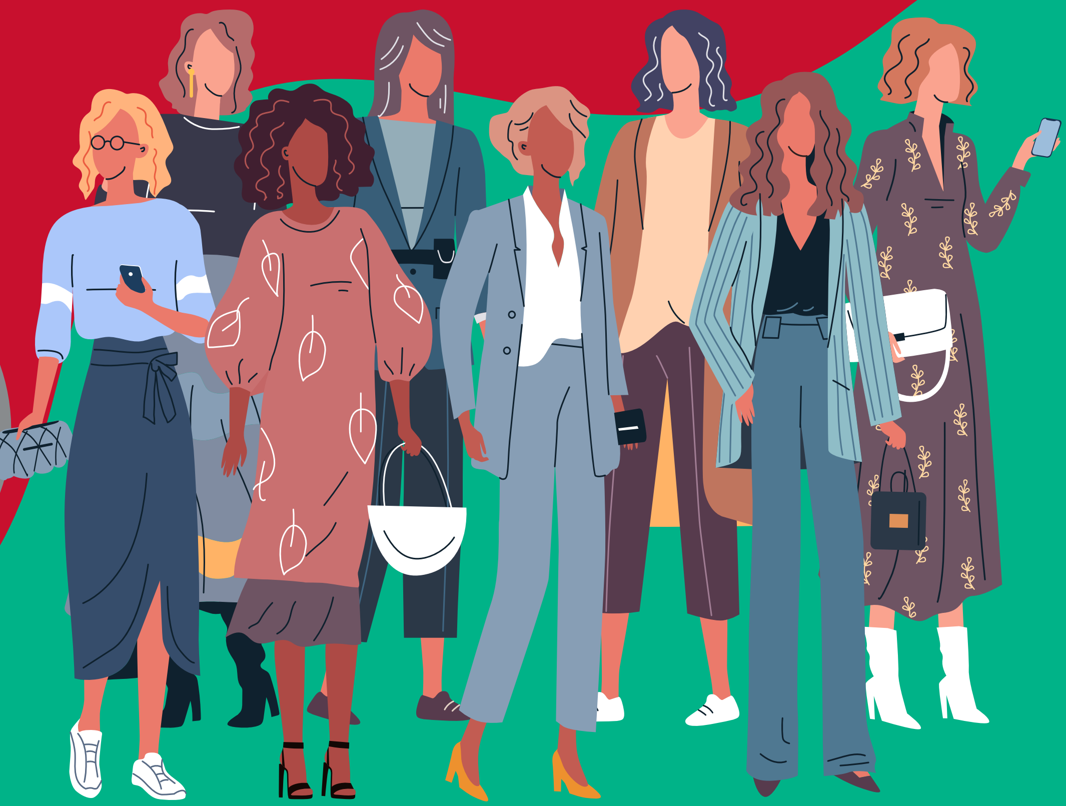

HEALTH
NIMHD – RCMI
Addictions Research and Cancer Prevention

UNIVERSITY OF HOUSTON

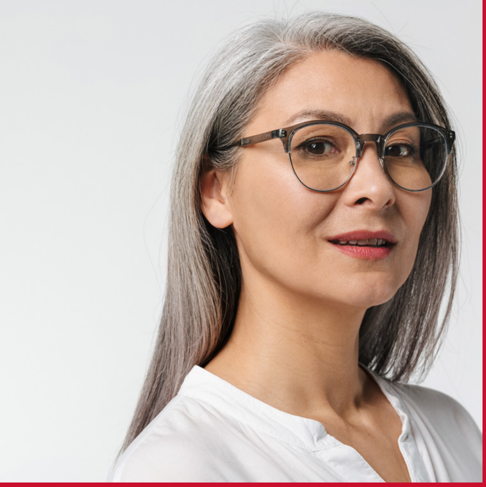

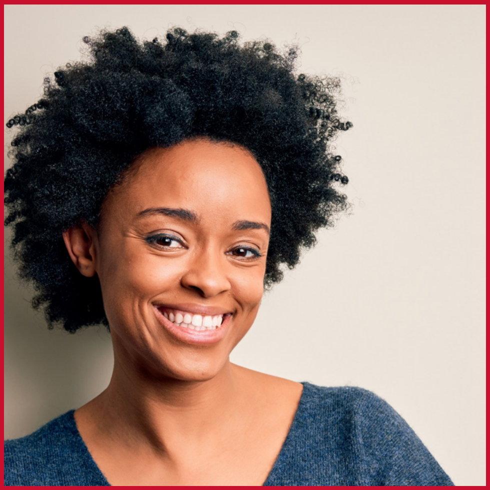

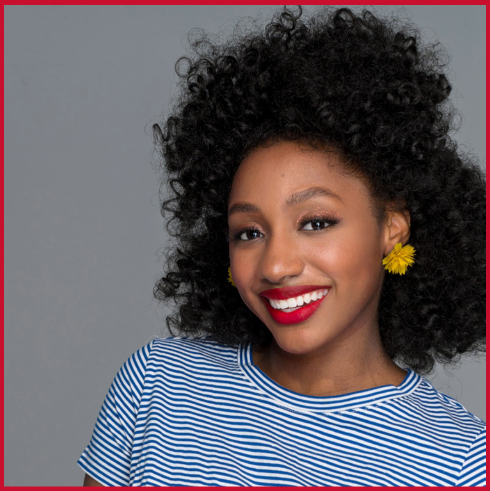

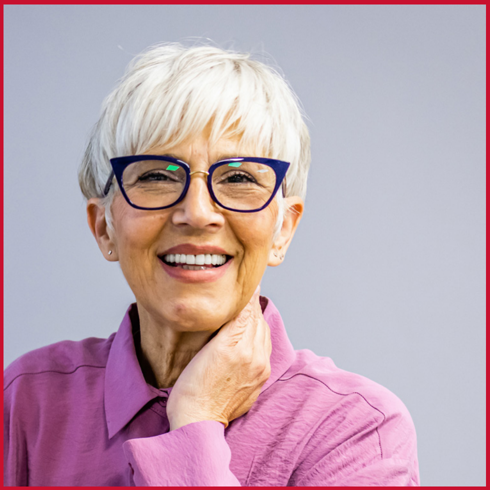

WOMEN SLEEP & MEMORY STUDY

Are you eligible to participate?

We are looking for minority women aged 30-65 that are right-handed, will take an online survey, and make two visits to our lab.

The study consists of:

- Responding to verbal cues during manual tasks
- Testing your cognitive functioning and memory
- Undergo a blood draw and body scan
- Evaluate your sleep characteristics

Qualified Participants will receive an Amazon gift card for completing:

- Online survey - $10
- First lab visit - $20
- Second lab visit - $60
- Sleep study - $200

Totaling $290 for attending all study visits.

SCAN HERE FOR ELIGIBILITY

For information on eligibility for this study:

Contact us by phone at 713.743.4802
Email us at sgorniak@uh.edu

HEALTH
NIMHD – RCMI
Addictions Research and Cancer Prevention

UNIVERSITY OF HOUSTON
